# Comprehensive landscape and interference of clonal haematopoiesis mutations for liquid biopsy: A Chinese pan‐cancer cohort

**DOI:** 10.1111/jcmm.16966

**Published:** 2021-10-17

**Authors:** Enwu Xu, Kai Su, Yang Zhou, Longlong Gong, Yiwen Xuan, Ming Liao, Jiawang Cao, Yaqian Li, Yujiao Lu, Yi Zhao, Fengxia Chen

**Affiliations:** ^1^ The First School of Clinical Medicine Southern Medical University Guangzhou China; ^2^ Department of Thoracic Surgery General Hospital of Southern Theater Command PLA Guangzhou China; ^3^ Genecast Biotechnology Co., Ltd Wuxi China; ^4^ Department of Thoracic Surgery Hainan General Hospital Haikou China

**Keywords:** cell‐free DNA, Chinese pan‐cancer cohort, clonal haematopoiesis, liquid biopsy, somatic mutations

## Abstract

Tumour‐derived DNA found in the plasma of cancer patients provides the probability to detect somatic mutations from circulating cell‐free DNA (cfDNA) in plasma samples. However, clonal hematopoiesis (CH) mutations affect the accuracy of liquid biopsy for cancer diagnosis and treatment. Here, we integrated landscape of CH mutations in 11,725 pan‐cancer patients of Chinese and explored effects of CH on liquid biopsies in real‐world. We first identified 5933 CHs based on panel sequencing of matched DNA of white blood cell and cfDNA on 301 genes for 5100 patients, in which CH number of patients had positive correlation with their diagnosis age. We observed that canonical genes related to CH, including *DNMT3A*, *TET2*, *ASXL1*, *TP53*, *ATM*, *CHEK2* and *SF3B1*, were dominant in the Chinese cohort and 13.29% of CH mutations only appeared in the Chinese cohort compared with the Western cohort. Analysis of CH gene distribution bias indicated that CH tended to appear in genes with functions of tyrosine kinase regulation, PI3K‐Akt signalling and TP53 activity, suggesting unfavourable effects of CH mutations in cancer patients. We further confirmed effect of driver genes carried by CH on somatic mutations in liquid biopsy of cancer patients. Forty‐eight actionable somatic mutations in 17 driver genes were considered CH genes in 92 patients (1.80%) of the Chinese cohort, implying potential impacts of CH on clinical decision‐making. Taken together, this study exhibits strong evidence that gene mutations from CH interfere accuracy of liquid biopsies using cfDNA in cancer diagnosis and treatment in real‐world.

## 1 INTRODUCTION

Tumour‐derived DNA was found in the plasma of cancer patients by Stroun et al.^1^ 30 years ago, providing the probability to detect somatic mutations from circulating cell‐free DNA (cfDNA) in plasma samples collected noninvasively. In addition to the advances in sequencing technology, somatic mutations detected from cfDNA have been used to diagnose and manage cancer patients in several approaches,^2^ including screening for early carcinoma,^3^ guiding systemic therapy^4^ and monitoring minimal residual disease (MRD).^5^ However, the origin of mutations observed in cfDNA from plasma samples is diverse and includes tumour‐ and clonal hematopoiesis (CH)‐derived mutations. Several studies have indicated that the source of some gene mutations in cfDNA is not the matched tumour in metastatic breast cancer (MBC), non‐small‐cell lung cancer (NSCLC) and castration‐resistant prostate cancer (CRPC).^6^, ^7^ These alterations are mainly due to interference from CH and have been proven by many scientists.^6^, ^7^, ^8^, ^9^ Thus, CH mutations in plasma can affect the accuracy of liquid biopsy for cancer diagnosis, treatment and management.

Although three genes, *DNMT3A*, *ASXL1* and *TET2*, are hot genes,^10^ CH mutations also appear in other genes, particularly some driver genes that are indicators for cancer therapy. Hu et al.^7^ found that 2 out of 58 (3.4%) advanced NSCLC patients with mutant *EGFR* had CH mutations in *KRAS* (G12X) that were persistent in the blood. Mutations in *KRAS* are genomic markers indicating resistance to tyrosine kinase inhibitors (TKIs) targeted to *EGFR*; thus, CH mutations in *KRAS* can lead to a misleading prognosis of TKIs. In addition to *KRAS*, several CH mutations in *JAK2* and *TP53* were also observed in patients with advanced NSCLC in this study. Furthermore, Jensen et al.^11^ found that 7 out of 69 (10.1%) patients with advanced prostate cancer had CH mutations in DNA repair genes, including *ATM*, *BRCA2* and *CHEK2*. Mutations in these DNA repair genes have been approved to determine the usage of poly (ADP‐ribose) polymerase inhibitor (PARPi) for patients with advanced prostate cancer, where CH mutations might induce misdiagnosis. Furthermore, Li et al.^12^ analysed the association between CH mutations and genomic markers related to immunotherapy. In particular, patients with a high tumor mutational burden (TMB) possessed CH mutations, implying probable effects brought by the improper counting of CH mutations. Taken together, CH mutations should be distinguished from tumour‐derived mutations in the guidance of various cancer treatments, regardless of targeted therapy or immunotherapy.

Studies focused on the relationship between CH mutation and cancer showed that an increased risk of haematologic cancers is associated with the existence of CH mutations,^10^, ^13^, ^14^ particularly those harboured by leukaemia driver genes (e.g. *DNMT3A*, *ASXL1*, *TET2*, *PPM1D*, *TP53*, *RAD21*, *STAG2*, *ATM*, *NF1*, *CALR*, *JAK2*, *CBL*, *SETD2* and *MPL*). Patients with CH mutations also had adverse prognoses of nonhaematologic cancers with shorter survival times,^13^ likely due to interactions between CH clones and cancer cells.^15^ Cancer therapy (such as radiotherapy or chemotherapy with platinum agents) can select CH clones with mutations in DNA damage response genes (including *TP53*, *PPM1D* and *CHEK2*), where the treatment dose is positively correlated with the enrichment of these CH mutations.^14^ Thus, the relationships among CH mutations, cancer progression and cancer treatment are complex.

Methods used to identify CH mutations based on panel sequencing data at approximately 1000× depth are different among studies. Coombs et al.^13^ called mutations from patient‐paired plasma and tumour samples; if the variant allele fraction (VAF) of a mutation in the plasma was greater than twice the VAF in the tumour, a CH mutation was determined. Li et al.^12^ and Bolton et al.^14^ followed the same method to identify CH mutations. However, this method could not completely exclude somatic mutations because of tumour heterogeneity. Jensen et al.^11^ defined CH mutation as a variant with VAFs of at least 2% in both samples of whole blood and plasma that was not observed in the matched tumour sample. Although sequencing of the matched tumour sample excluded germline mutations, the latter could not be excluded completely because of loss of heterozygosity (LOH) events in the tumour. Thus, the method for CH mutation identification based on panel sequencing data should be improved. Another study adopted high‐intensity sequencing with a depth larger than 60,000× for cfDNA and matched peripheral blood lymphocyte (PBL) samples and machine learning models to call CH mutations.^6^ Ultra‐deep sequencing elevated both the sensitivity and specificity in CH mutation calling, resulting in a much more accurate and clearer CH landscape. In our study, we compared the CH landscape between the Chinese and Western cohorts.

In this study, we comprehensively profiled CH mutations using a bioinformatics pipeline and revealed the characterization of CH mutations in a Chinese pan‐cancer cohort. To improve the accuracy of CH mutation detection, we identified CH mutations under a statistical framework to compare the distribution of alteration‐supporting reads in cfDNA and matched PBL samples using Fisher's exact test while considering mutations whose distribution of alteration‐supporting reads was similar in both samples as CH mutations. To further avoid false‐positive calling, we filtered out CH mutations that appeared in matched tumour samples. Next, we systematically compared the landscape of CH mutations between the Chinese and Western cohorts discussed above. We further defined CH‐, germline‐ and somatic‐preferred genes according to the distribution of CH, germline and somatic mutations and found distinctive patterns of enriched functions for different categories. Additionally, we evaluated the potential effects of CH mutations on liquid biopsy, suggesting that some risk exists from CH in the diagnosis of tumours and administration of targeted anticancer drugs in the real world.

## 2 MATERIALS AND METHODS

### 2.1 Sample collection

In total, 11,725 pan‐cancer patients and 30 asymptomatic individuals without known cancer were enrolled in this study. For all cancer patients, the genomic DNA of PBL and cfDNA in plasma were extracted and sequenced to identify CH mutations. The tumour tissues of 2336 cancer patients were also collected for sequencing to validate the true CH mutations. For asymptomatic individuals, the genomic DNA of PBL and cfDNA in plasma were extracted and sequenced to identify CH mutations.

### 2.2 DNA extraction

The genomic DNA of PBL was extracted using the TGuide S32 Magnetic Blood Genomic DNA Kit (Tiangen). cfDNA in plasma was extracted using the MagMAX Cell‐Free DNA (cfDNA) Isolation kit (Thermo Fisher Scientific). The genomic DNA of tumour tissue was extracted from formalin‐fixed paraffin‐embedded (FFPE) samples using the MagPure FFPE DNA Kit B (Magen). The concentration of extracted DNA was measured using the Qubit dsDNA HS (High Sensitivity) Assay Kit (Thermo Fisher Scientific), and the quality of extracted DNA was assessed using the Agilent 2100 BioAnalyzer (Agilent).

### 2.3 Library preparation

The genomic DNA from PBL was fragmented into DNA pieces of approximately 200 base pairs (bp) using an enzymatic method (5×FEA Enzyme Mix; Qiagen). After end repair and A tailing, T‐adaptors were ligated on both ends (TIANSeq DNA Ligase, Qiagen), and final libraries were obtained after PCR amplification (10×KAPA Library Amplification Primer Mix; KAPA Biosystems). Genomic DNA from FFPE samples was sheared into 150‐ to 200‐bp fragments using the Covaris M220 Focused‐ultrasonicator™ Instrument (Covaris) according to the recommended settings. Fragmented genomic DNA from FFPE and cfDNA libraries was constructed using a VAHTS Universal DNA Library Prep Kit (Vazyme) following the manufacturer's instructions. All the libraries were quantified using an AccuGreen High Sensitivity dsDNA Quantitation Kit (Biotium), while the size of the libraries was determined using an Agilent Bioanalyzer 2100 system (Agilent).

### 2.4 Capture of targeted regions and sequencing

For cancer patients, the libraries of genomic DNA and cfDNA were captured using an in‐house designed panel spanning a 1.89‐Mb genomic region and including 468 genes (Table S3). For asymptomatic individuals, the libraries of genomic DNA and cfDNA were captured using another in‐house‐designed panel spanning a 0.55‐Mb genomic region and including 118 genes (Table S4). Capture of the targeted regions was performed using a HyperCap Target Enrichment Kit (Roche). The hybridization and washing steps were conducted according to the manufacturer's protocol. Next, the captured libraries were sequenced using the NovaSeq 6000 system (Illumina) according to the manufacturer's protocol, producing paired‐end reads with a 150‐bp length at each end. For cancer patients, genomic DNA extracted from PBL was sequenced at a depth of at least 200×, while cfDNA extracted from plasma and genomic DNA extracted from FFPE tumour samples were sequenced at a depth of at least 1000×. For asymptomatic individuals, genomic DNA extracted from PBL and cfDNA extracted from plasma were sequenced with mean depths of 1723× and 2383×, respectively.

### 2.5 Data processing and mutation calling

Adaptor sequences and low‐quality bases of sequenced reads were trimmed using Trimmomatic (v0.36)^16^ to obtain clean reads. Clean reads were mapped to the human reference genome (hg19) using BWA (v0.7.17).^17^ The mapping results were sorted and masked for duplications using Picard (v2.23.0).^6^ From the sorted and duplication‐masked mapping results of PBL, cfDNA and tumour tissue samples, SNVs and InDels were called using VarDict (v1.5.1),^18^ while complex mutations were called using FreeBayes (v1.2.0).^19^ To avoid false‐positive results, SNVs and InDels that appeared in the blacklist (including sequence‐specific errors, repeat regions, segmental duplications and lowly mappable regions recorded in ENCODE^20^) were removed. The filtered mutations were annotated using ANNOVAR (2015Jun17),^21^ and synonymous mutations were not considered in this study.

### 2.6 Identification of somatic, germline and CH mutations

First, mutations detected only in cfDNA were considered candidate somatic mutations. We retained somatic mutations that satisfied the following criteria: (1) the sequencing depth of the mutation was not smaller than 100×; (2) the number of reads supporting the variant allele was not smaller than 2; (3) the VAF of the mutation was not smaller than 0.3% and (4) the minor allele frequency (MAF) of the mutation in the gnomAD^22^ and ExAC^23^, ^24^ databases was not larger than 0.2%. Furthermore, mutations recorded in the dbSNP^25^ database but not in the COSMIC^26^, ^27^ database and mutations in the HLA locus were filtered out. Second, mutations present in PBL with a sequence depth of at least 30× and VAF not smaller than 20% were considered candidate germline mutations. In the subsequent analysis, we retained only pathogenic germline mutations that were recorded as ‘Pathogenic’ or ‘Likely pathogenic’ in the ClinVar database.^28^ Third, for mutations present in PBL with a sequence depth of at least 30× but VAF smaller than 20%, we conducted Fisher's exact test on the count distribution of reference and variant alleles in paired samples of cfDNA and PBL to identify CH mutations. If the *p*‐value was not smaller than 0.05 and the odds ratio was larger than 0.5 and smaller than 1.5, the mutation was considered a candidate CH mutation. Otherwise, the mutation was considered a candidate somatic (variant alleles appeared much more frequently in the cfDNA sample, the *p*‐value was smaller than 0.05, and the odds ratio was larger than (1). Candidate CH mutations were retained if there were no fewer than 2 reads supporting the variant allele in both cfDNA and PBL samples. Then the carrier ratio of each CH was obtained by calculating the percentage of samples carrying each mutation in total samples carrying at least one CH mutation. We further filtered out CH mutations whose carrier ratio was larger than 0.25% or that were called somatic mutations from the matched tissue sample.

### 2.7 Correlation analysis between CH mutation and diagnostic age

We grouped the pan‐cancer patients according to the range of diagnostic age, including ≤40, 40–50, 50–60, 60–70, 70–80, 80–90 and 90–100 years. For each group, we calculated the percentage of patients carrying at least one CH mutation (Table S5). Pearson's correlation coefficient between the percentage and age group was calculated. For each cancer type, we conducted the same analysis.

### 2.8 Distribution difference of 189 genes in the Chinese and Western cohorts

After removing genes only included in the respective panels used for sequencing, we compared the distribution of CH mutations in 277 genes between Chinese and Western cohorts and found that 189 of those genes carried CH mutations in both cohorts. We counted the number of samples with and without CH mutations in these genes in the Chinese and Western cohorts, and used Fisher's exact test to determine whether any genes tended to be enriched in one of the cohorts. The odds ratio (OR = [a × d]/[b × c]) and two‐tailed *p*‐value was calculated with the 2 × 2 table as bellow:

**Table:** 

	Chinese cohorts	Western cohorts
Sample number with CH mutations in genes	a	b
Sample number without CH mutations in genes	c	d

### 2.9 Identification of CH‐, germline‐ and somatic‐preferred genes

We defined CH mutations that were also germline or somatic mutations in other patients as polymorphic CH mutations and selected them for this analysis. For each polymorphic CH mutation, we calculated its percentage to be CH, germline or somatic mutation in this cohort. If its percentage to be CH, germline or somatic mutation was larger than 50%, the polymorphic CH mutation was labelled as CH‐, germline‐ or somatic‐preferred mutation. Based on the labelled mutations, we considered genes with CH‐preferred mutations only as CH‐preferred genes, with CH‐preferred and more germline‐preferred than somatic‐preferred mutations as germline‐preferred genes while with CH‐preferred and more somatic‐preferred than germline‐preferred mutations as somatic‐preferred genes.

### 2.10 Overlap CH mutations with actionable mutations

We overlapped CH mutations with actionable mutations collected in a knowledge base constructed by Genecast Biotechnology Co., Ltd., such as germline and somatic mutations that are therapy targets or prognosis markers. Overlaps were called if the genomic position and variant allele were exactly matched between CH mutations and actionable mutations. Overlapping CH mutations were listed and used to conduct descriptive statistics analysis (Table S6).

### 2.11 Statistical analysis and data visualization

Functional enrichment analysis was conducted using Fisher's exact test using an in‐house R (version 3.6.1) script wrapped in the package of clusterProfiler^29^ (version v3.10.1) with annotations of Gene Ontology,^30^, ^31^ KEGG^32^ and Reactome^33^ databases. Genes enriched with CH mutations in Chinese or Western cohort were identified by using Fisher's exact test using an in‐house R script. Multiple testing correction was conducted using the FDR method using an in‐house R script. The mutational landscape was visualized using the R package ComplexHeatmap,^34^ while the distribution of CH mutations in genes was plotted using the R package maftools.^35^

## 3 RESULTS

### 3.1 Identification of CH mutations in cancer patients

**FIGURE 1:**
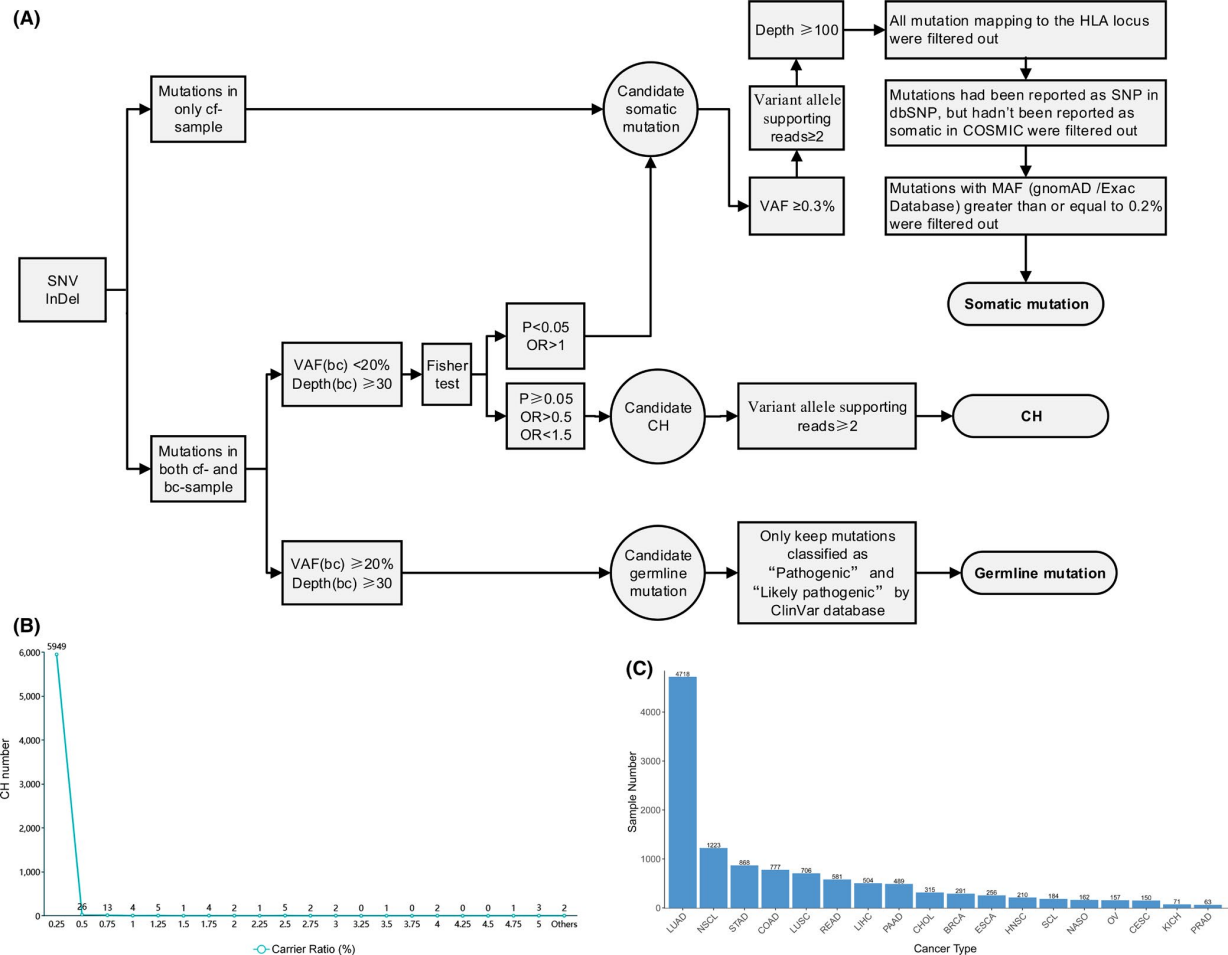
Workflow of screening CH mutations. (A) Variation screen pipeline. (B) Carrier ratio of candidate CH mutations. The *X* axis is the carrier ratio of each candidate CH mutation, and the *Y* axis is the sample number equal to or less than the corresponding carrier ratio. The carrier ratio was obtained by calculating the percentage of samples carrying each mutation in total samples carrying at least one CH mutation. (C) Distribution of the sample number. BRCA, breast invasive carcinoma; CH, clonal hematopoiesis; CESC, cervical squamous cell carcinoma and endocervical adenocarcinoma; CHOL, cholangiocarcinoma; COAD, colon adenocarcinoma; ESCA, esophageal cancer; HNSC, head and neck squamous cell carcinoma; KICH, kidney chromophobe; LIHC, liver hepatocellular carcinoma; LUAD, lung adenocarcinoma; LUSC, lung squamous cell carcinoma; NASO, nasopharyngeal carcinoma; NSCLC, non‐small‐cell lung carcinoma; OV, ovarian serous cystadenocarcinoma; PAAD, pancreatic adenocarcinoma; PRAD, prostate adenocarcinoma; READ, rectum adenocarcinoma; SCL, small cell lung cancer; STAD, stomach adenocarcinoma

By comparing the alteration‐supporting reads distributed in the genomic DNA of PBL and matched cfDNA extracted from plasma samples, we comprehensively identified CH mutations using an in‐house pipeline (Figure 1A; see Methods for details). If no distribution bias of alteration‐supporting reads was found for a specific mutation (*p*‐value reported by Fisher's exact test was not smaller than 0.05), we called it as CH mutation. In total, we called 6034 candidate CH mutations. Next, we adopted several criteria to filter out candidate CH mutations. First, we filtered out candidate CH mutations whose number of alteration‐supporting reads was smaller than 2 in both PBL and matched plasma samples, with 6023 remaining. Second, considering that the generation of CH mutations was random in populations, we filtered out candidate CH mutations whose carrier ratio was larger than 0.25%. We selected 0.25% as the carrier ratio threshold because we observed that most of the candidate CH mutations (5949 of 6023; 98.8%) had that carrier ratio (Figure 1B). However, 74 candidate CH mutations whose carrier ratio was larger than 0.25%, suggesting that those mutations might be technical artefacts and should be removed. Third, to further avoid false‐positive calling, we removed 16 candidate CH mutations that were detected as somatic mutations in matched tumour tissue. The low proportion of tissue‐observed candidate CH mutations (16 of 5949; 0.27%) revealed the robustness of this CH‐calling pipeline and underlying statistical framework. Finally, we identified 5933 CH mutations in 5100 pan‐cancer patients.

Next, we identified CH mutations for 30 asymptomatic individuals based on the sequencing data of paired samples of PBL and plasma using the same pipeline. After the filtering steps, 4 CH mutations were called for 4 of 30 (13.3%) asymptomatic individuals. To compare the prevalence of CH mutations in cancer patients and asymptomatic individuals, we counted CH mutations located in 118 genes (which appeared in the panel used to capture DNA for asymptomatic individuals) for cancer patients and found that 1698 of 11,725 (14.5%) patients carried at least one counted CH mutation, showing a similar prevalence between the two cohorts (odds ratio = 0.921; *p*‐value = 1 by Fisher's exact test).

### 3.2 Landscape of CH mutations in Chinese pan‐cancer patients

**FIGURE 2:**
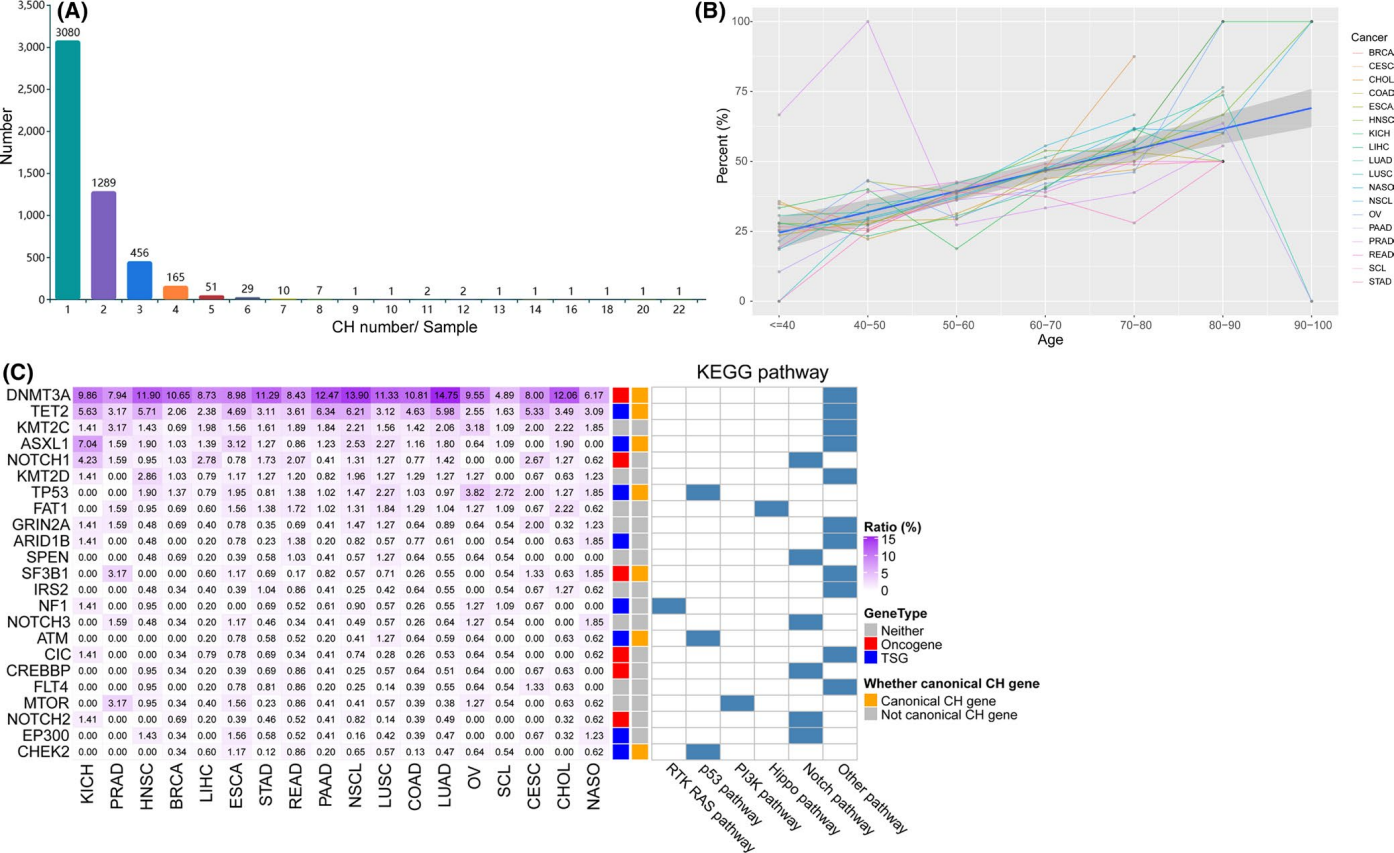
Characterization of clonal hematopoiesis (CH) mutations in the Chinese pan‐cancer cohort. (A) Distribution of pan‐cancer patients with different numbers of CH mutations. (B) Association between age and the CH carrier ratio. The *X* axis represents the age, and the *Y* axis represents the percentage of the sample number carrying at least one CH over the total sample number at the corresponding age. (C) Heatmap of the top mutated genes carrying CH in different cancer types according to genes by sample number. The number in the cells indicate ‘ratio’ for each gene in the corresponding cancer type. The ‘Ratio’ represents the carrier ratio, namely the percentage of samples that carried CH mutations in each gene and total samples of the corresponding cancer type

We identified CH mutations in a large Chinese pan‐cancer cohort, including 11,725 patients with 18 cancer types (Figure 1C). The mean diagnostic age of all the patients was 60 years (ranging from 13 to 96 years). In total, 5933 CH mutations in 301 genes were identified for 5100 patients (43.5% of the Chinese cohort; Table S1). Most patients possessed one (3080 of 5100 patients; 60.4%) or two CH mutations (1289 of 5100 patients; 25.3%; Figure 2A). The distribution of CH mutation number of patients was similar across different cancer types (Figure S1A; Table S2), while the mean number of CH mutations was 1.66 in all cancers, ranging from 1.41 in liver hepatocellular carcinoma (LIHC) to 1.94 in rectum adenocarcinoma (READ). Consistent with previous studies,^21^, ^22^ we observed a strongly positive correlation between the percentage of patients with CH mutations and diagnostic age across different cancer types (Pearson's correlation coefficient = 0.997; *p*‐value = 1.38 × 10^−6^, Figure 2B; Table 1), suggesting that CH occurred more frequently in older patients.

**TABLE 1:** Prevalence of clonal hematopoiesis (CH)

Age	Sample number carrying CH	Total sample number	Percent (%)
≤40	179	684	26.17
40–50	512	1685	30.39
50–60	1237	3207	38.57
60–70	1925	4015	47.95
70–80	1025	1795	57.10
80–90	217	332	65.36
>90	5	7	71.43

Next, we calculated the carrier ratio for genes harbouring CH mutations and ranked them according to the sample number carrying CH mutations (Figure 2C). For the top 20 genes in the ranking, 7 were canonical genes associated with CH—i.e., *DNMT3A*, *TET2*, *ASXL1*, *TP53*, *SF3B1*, *ATM* and *CHEK2*. As reported by previous study that CH mutations in epigenetic modifiers *DNMT3A*, *TET2* and *ASXL1* were more frequently observed in middle‐aged individuals,^36^ we found that these genes were hot genes enriched with CH mutations in this Chinese cohort. A total of 1457 (28.6%), 574 (11.3%) and 200 (3.9%) patients carried at least one CH mutation in *DNMT3A*, *TET2* and *ASXL1*, respectively. Along with the sample number, mutational sites of these genes were also much more than other genes (Figure S1B), with 925 (15.6% of all mutational sites), 507 (8.5%) and 161 (2.1%) mutational sites in *DNMT3A*, *TET2* and *ASXL1*, respectively. Notably, many more CH mutations in these genes were dysfunctional, among which 446 (48.2%), 357 (70.4%) and 140 (87%) CH mutations were frameshift insertions and deletions (InDels) or nonsense single‐nucleotide variants (SNVs) in *DNMT3A*, *TET2* and *ASXL1*, respectively, suggesting the potential harmful effects of CH mutations in these genes.

Within the top 20 genes in the ranking of sample number, there were 6 oncogenes (including *DNMT3A*, *NOTCH1*, *SF3B1*, *CIC*, *CREBBP* and *NOTCH2*) and 8 tumour suppressor genes (TSGs, including *TET2*, *ASXL1*, *TP53*, *ARID1B*, *NF1*, *ATM*, *EP300* and *CHEK2*). Additionally, 6 genes were enriched in the Notch pathway (including *NOTCH1*, *SPEN*, *NOTCH3*, *CREBBP*, *NOTCH2* and *EP300*), and 3 genes were enriched in the p53 pathway (*TP53*, *ATM* and *CHEK2*). Taken together, these observations indicated that CH mutations might impact both oncogenic and tumour‐suppressive functions.

### 3.3 Comparison of CH mutations with Western cohort

**FIGURE 3:**
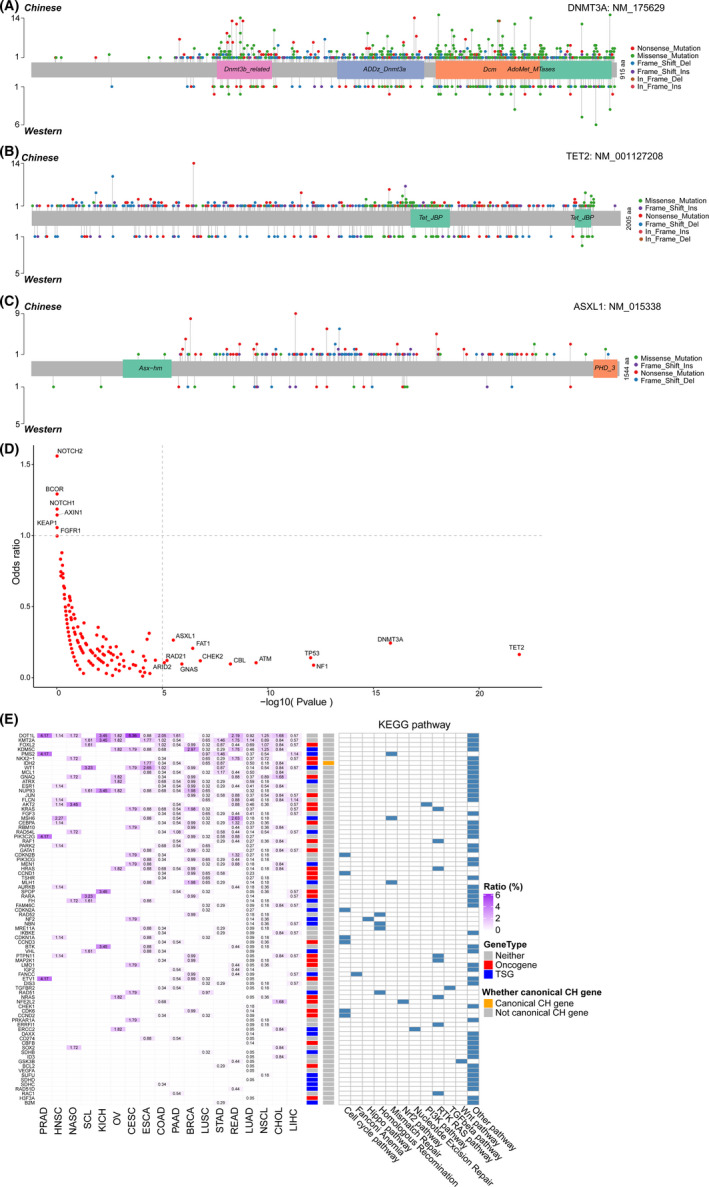
Differences in clonal hematopoiesis (CH) mutations between the Chinese and Western cohorts. (A‐C) Mutation comparison between the Chinese and Western cohorts. For all plots, the *Y* axis shows mutation counts, and the *X* axis depicts the position of variants with relevant domains noted in the inset for each gene. The distribution of *DNMT3A* (A), *TET2* (B) and *ASXL1* (C) mutations are showed between the Chinese (top) and Western (bottom) cohorts. (D) Distribution difference of common genes in the Chinese and Western cohorts. The *X* axis represents the *p* value calculated by Fisher's exact test, and the *Y* axis represents the corresponding odds ratio. Only the 189 genes carrying CH mutations detected in both the Chinese and Western cohorts are considered. (E) Heatmap of 81 genes is identified exclusively in the Chinese cohort

To detect whether a distinctive pattern exists, we compared the landscape of CH mutations between our Chinese cohort and a Western cohort.^19^ After removing genes only included in the respective panels used for sequencing, we compared the distribution of CH mutations in 277 genes between these two cohorts and found that 189 of those genes carried CH mutations in both cohorts. First, we observed that the *DNMT3A*, *TET2* and *ASXL1* genes were hot genes of CH mutations in the Western cohort, consistent with that in the Chinese cohort. Based on depicting the domain distribution of CH mutations in those genes, we found that the pattern was similar between the Chinese and Western cohorts (Figure 3A‐C), implying similar functional effects of these CH mutations. Second, we found no evident gene enrichment of CH mutations in the Chinese cohort compared with that in the Western cohort (Figure 3D), although several tumour suppressor genes involved in the Wnt pathway (*AXIN1*), Nrf2 pathway (*KEAP1*) and Notch pathway (*NOTCH1* and *NOTCH2*) showed the potential of enrichment. By contrast, we found that CH mutations were significantly enriched in tumour suppressor genes involved in the p53 pathway (*TP53*, *ATM* and *CHEK2*), RTK/RAS pathway (*NF1* and *CBL*) and Hippo pathway (*FAT1*) and genes functioning in epigenetic modification (*DNMT3A*, *TET2* and *ASXL1*) and DNA double‐strand break repair (*RAD21*) in the Western cohort (Figure 3D). Third, we observed that 81 genes carried CH mutations exclusively in the Chinese cohort (27 oncogenes and 23 tumour suppressor genes; Figure 3E), whereas only 2 genes (*REL* and *IKZF1*) carried CH mutations exclusively in the Western cohort. Notably, genes with CH mutations exclusively in the Chinese cohort were mainly involved in the RTK/RAS pathway (8 of 81; 9.9%), cell cycle pathway (7 of 81; 8.6%), homologous recombination pathway (4 of 81; 4.9%) and mismatch repair pathway (3 of 81; 3.7%).

### 3.4 Functional bias of CH‐, germline‐ and somatic‐preferred genes

We found that CH mutations in a patient might be simultaneously germline or somatic mutations in others for this Chinese cohort. Thus, we classified genes into three categories according to the percentage of CH, germline and somatic mutations appeared in genes, including CH‐preferred (*n* = 256), germline‐preferred (*n* = 0), and somatic preferred (*n* = 38) genes (see Methods for details).

**FIGURE 4:**
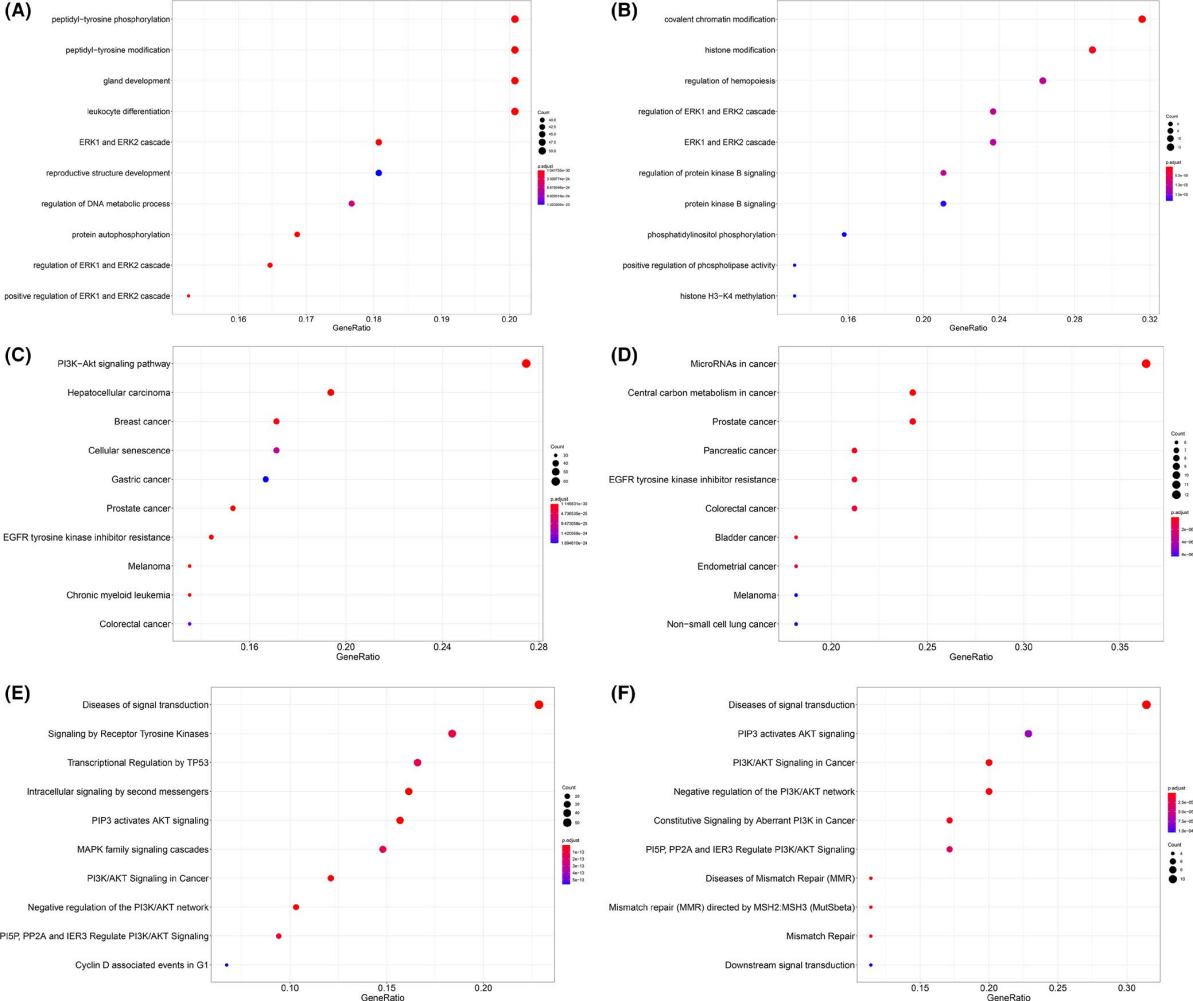
Functional enrichment analysis for different gene sets. (A and B) GO enrichment (biological process) of clonal hematopoiesis (CH)‐preferred (A) and somatic‐preferred (B) genes. (C and D) KEGG enrichment of CH‐preferred (C) and somatic‐preferred (D) genes. (E and F) Reactome enrichment of CH‐preferred (E) and somatic‐preferred (F) genes

Next, we conducted functional enrichment analysis to profile functional bias for genes in each category. First, CH‐preferred genes were enriched in leukocyte differentiation (GO; Biological Process; odds ratio = 8.93; Fisher's exact test; FDR = 3.84 × 10^−26^), transcriptional regulation by *TP53* (Reactome; odds ratio = 5.54; Fisher's exact test; FDR = 1.31 × 10^−13^) and RTK/RAS‐related cascades, such as *EGFR* tyrosine kinase inhibitor resistance (KEGG; odds ratio = 17.09; Fisher's exact test; FDR = 3.69 × 10^−28^), *ERK1* and *ERK2* cascade (GO; Biological Process; odds ratio = 11.65; Fisher's exact test; FDR = 2.02 × 10^−28^), MAPK family signalling cascades (Reactome; odds ratio = 6.20; Fisher's exact test; FDR = 1.31 × 10^−13^) and PI3K‐Akt signalling pathway (KEGG; odds ratio = 8.28; Fisher's exact test; FDR = 1.15 × 10^−30^) (Figure 4A,C,E). Second, somatic‐preferred genes were also enriched in RTK/RAS‐related cascades as CH‐preferred genes and were uniquely enriched in diseases of mismatch repair (MMR) (Reactome; odds ratio = 226.99; Fisher's exact test; FDR = 6.91 × 10^−7^) and mismatch repair (MMR) directed by *MSH2*:*MSH3* (Reactome; odds ratio = 90.72; Fisher's exact test; FDR = 1.03 × 10^−5^) (Figure 4B,D,F). Distinctive functional bias indicated that CH mutations in genes of different categories had different influences on the haematopoiesis of cancer patients.

### 3.5 Interference of CH mutations on liquid biopsy

**FIGURE 5:**
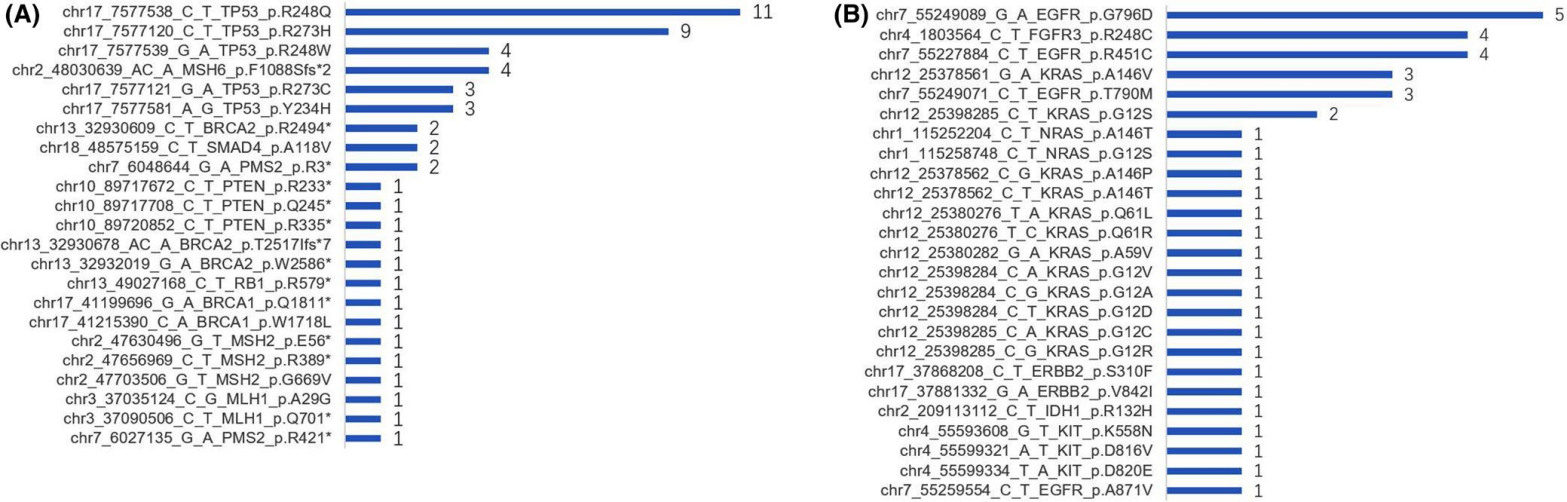
Effects of clonal hematopoiesis (CH) mutations on liquid biopsies in clinical diagnosis. (A‐B) Forty‐eight clinically significant somatic mutations are found in 17 driver genes, including 7 oncogenes and 10 tumour suppressor genes. The number of specific mutations are showed in 10 tumour suppressor genes (A) and 7 oncogene genes (B)

Liquid biopsy is a common strategy to detect actionable somatic mutations, which are therapy targets or prognosis markers, for cancer patients. However, the existence of CH mutations in plasma affects the power of identifying true actionable somatic mutations. To uncover the scope of influence caused by CH mutations, we overlapped them with actionable somatic mutations and found that 48 of 5933 (0.81%) CH mutations carried by 92 of 5100 (1.8%) cancer patients were also actionable somatic mutations. These 48 CH mutations were distributed in 10 TSGs (including *BRCA1*, *BRCA2*, *MLH1*, *MSH2*, *MSH6*, *PMS2*, *PTEN*, *RB1*, *SMAD4* and *TP53*) (Figure 5A) and 7 oncogenes (including *EGFR*, *ERBB2*, *FGFR3*, *IDH1*, *KIT*, *KRAS* and *NRAS*) (Figure 5B). We found that overlaps between CH mutations and actionable somatic mutations occurred much more frequently in the genes *TP53* (30 of 92 cancer patients, 32.6%; Fisher's exact test; FDR = 1.68 × 10^−22^) and *KRAS* (15 of 92 cancer patients, 16.3%; Fisher's exact test; FDR = 7.25 × 10^−19^). By summarizing the mutations and clinical characteristics of patients, we found that CH mutations in *KRAS* occurred most frequently in patients with NSCLC (8/15; 53.3%).

The presence of *KRAS/NRAS* mutations suggests the lack of response to *EGFR*‐targeted therapies for patients with NSCLC, head and neck cancer or colorectal cancer. In this study, 14 mutations carried by 16 patients were found in exon 2/3/4 of *KRAS* and *NRAS*. Among these patients, one with NSCLC and one with colorectal cancer might lose the chance of treatment with cetuximab or panitumumab despite also carrying *EGFR* mutations. Additionally, 6 cases were identified with lung/ovarian cancer carrying CH mutations in *BRCA1/2* that might be particularly sensitive to PARP inhibitors such as olaparib, rucaparib and talazoparib. Additionally, 6 stop‐gain and 2 nonsynonymous CH mutations in *MLH1*, *MSH2* and *PMS2* that might lead to mismatch repair deficiency (dMMR) were identified in 8 patients, and these confusions would result in unsuccessful immunotherapy using pembrolizumab. In addition to the mutations mentioned above, we also found CH mutations in genes playing important roles in cancer therapy, such as *FGFR3*, *EGFR*, *PTEN*, *ERBB2* and *TP53*, indicating the importance of correctly managing CH mutations when making clinical decisions based on liquid biopsy.

## 4 DISCUSSION

Although liquid biopsy has largely been used to identify gene mutation sites and guide anticancer drugs by applying NGS technologies, CH‐associated mutations from peripheral blood might interfere with the detection results in solid tumours.^7^, ^37^ In this study, we screened CH mutation sites and explored the characterization of CH mutations in 18 different cancer types from Chinese populations. Among the top mutated genes, 6 oncogenes (*DNMT3A*, *NOTCH1*, *SF3B1*, *CIC*, *CREBBP* and *NOTCH2*) and 8 TSGs (*TET2*, *ASXL1*, *TP53*, *ARID1B*, *NF1*, *ATM*, *EP300* and *CHEK2*) were contained. The oncogenes *NOTCH1*, *NOTCH2* and *CREBBP* and TSG *EP300* belong to the Notch pathway. Aberrant Notch signalling is frequently reported in different cancers, such as breast cancer,^38^ lung cancer,^39^ colon cancer^40^ and adenoid cystic carcinoma.^41^ Recent research indicates that Notch signalling inhibitors, such as MRK‐003 and BMS‐906024, suppress tumour proliferation and migration.^42^ Additionally, *KRAS* and *TP53* CH mutations, which might also disturb the diagnosis of somatic gene mutations, are often observed in NSCLC in this study. Thus, identifying CH mutations, which are specifically related to anticancer target drugs, excludes the use of unsuitable drugs in clinical cancer treatment.

We found that 13 canonical genes in the Chinese cohort (*PPM1D* and *SRSF2* were not captured in our panel) among the genes containing DNA mutations associated with CH. In total, 1897 mutations lied on the 13 canonical genes^6^ known to be associated with CH,^6^ accounting for 32% (1897 of 5933) of all detected mutations. The *DNMT3A*, *TET2* and *ASXL1* genes had the most mutation sites in different cancer types from the Chinese populations. Similarly, the *DNMT3A*, *TET2*, *PPM1D*, *TP53* and *ASXL1* genes represent most of the CH mutation genes in MBC, NSCLC and CRPC from the American cohort.^6^ CH mutations in the *DNMT3A*, *TET2*, and *ASXL1* genes are frequently observed in individuals after middle age.^36^ We further revealed that the CH mutation spectrum in the *DNMT3A*, *TET2*, and *ASXL1* genes was similar between the Chinese and Western cohorts. However, 81 genes were uniquely identified in the Chinese cohort, and only 2 genes were uniquely identified in the Western cohort. This result might be due to differences in sequencing depth and sample size in the Chinese and Western cohorts.

The haematopoietic system is responsible for approximately one trillion (10^12^) cells arising daily in the adult human bone marrow.^43^ The genetic diversity within the haematopoietic stem cell compartment is significant in aged individuals, and each haematopoietic stem cell may acquire on the order of one exotic somatic mutation per decade.^44^ Our data showed that CH‐preferred genes were enriched in cell proliferation and metabolic regulation (such as the PI3K/Akt signalling pathway), suggesting that these genes are active and working. A previous report indicated that 10% of persons older than 65 years, but only 1% of those younger than 50 years, had CH mutations,^10^ revealing that CH mutation is increasingly common as individuals age.^10^, ^45^ The positive correlation between CH and age is consistent with the age‐associated decrease in DNA repair capacity.^46^, ^47^ Thus, our data showed that somatic‐preferred genes are enriched in many signalling pathways related to DNA mismatch repair, implying that CH mutations in somatic‐preferred genes promotes the generation of CH mutations in PBLs.

The NCCN Clinical Practice Guidelines recommend that if insufficient tissue is available to allow molecular testing, repeat biopsy and/or blood testing should be performed. In the real world, liquid biopsy has been more frequently used in clinical diagnosis and treatment. Because of tissue heterogeneity, in some cases, plasma testing can reveal more mutation information than tissue testing. However, the existence of CH mutations in cfDNA would affect the accuracy of liquid biopsy. We found 48 clinically significant mutations in 17 genes among 5100 patients derived from CH, not tumours. Among them, the *TP53* and *KRAS* genes were the most common CH genes. Several case reports are currently available concerning CH mutations in *KRAS*; whether these mutations affect the efficacy of the patient's targeted therapies requires further research data. Therefore, both plasma and PBL samples must be detected to distinguish tumour‐derived mutations and CH mutations, preventing misdiagnosis and mistreatment.

## 5 CONCLUSIONS

This study helped to characterize CH mutations in a large Chinese pan‐cancer cohort and compared the landscape of CH mutations between the Chinese and Western cohorts. Heterogeneity in the different types of CH genes was also explored. We further investigated overlap between CH mutations and drug‐targeted somatic mutations in clinical examination. In summary, our research provides additional strong evidence that CH mutations can interfere with the liquid biopsy of plasma for cancer diagnosis and treatment.

## CONFLICT OF INTEREST

Five of the authors (YZ, LG, YL, YL and YZ) affiliated with Beijing Genecast Biotechnology Co., Ltd performed next generation sequencing of ctNDA and analysis of data. The other authors declare that they have no competing interests.

## AUTHOR CONTRIBUTIONS

**Enwu Xu:** Conceptualization (lead); Formal analysis (equal); Investigation (equal); Project administration (equal); Supervision (equal); Validation (equal); Visualization (equal); Writing‐review & editing (equal). **Kai Su:** Formal analysis (equal); Visualization (equal); Writing‐review & editing (equal). **Yang Zhou:** Formal analysis (equal); Investigation (equal); Software (equal); Writing‐original draft (equal). **Longlong Gong:** Formal analysis (equal); Investigation (equal); Visualization (equal); Writing‐original draft (equal). **Yiwen Xuan:** Formal analysis (supporting); Investigation (supporting); Resources (equal). **Ming Liao:** Formal analysis (supporting); Investigation (supporting); Resources (equal). **Jiawang Cao:** Formal analysis (supporting); Investigation (supporting); Resources (equal). **Yaqian Li:** Formal analysis (supporting); Software (supporting); Visualization (supporting). **Yujiao Lu:** Software (supporting); Validation (supporting); Visualization (supporting). **Yi Zhao:** Formal analysis (supporting); Software (supporting); Writing‐review & editing (lead). **Fengxia Chen:** Conceptualization (equal); Formal analysis (equal); Supervision (equal); Writing‐review & editing (equal).

## PATIENT CONSENT STATEMENT

Written informed consent was obtained from all patients participating in the study for use of the samples for research and publication.

## Supporting information

Figure S1

Table S1‐6

## Data Availability

The datasets are available from the corresponding author and Genecast Biotechnology Co., Ltd on reasonable request.
