# Supplementary material for: Comprehensive landscape and interference of clonal haematopoiesis mutations for liquid biopsy: A Chinese pan‐cancer cohort
Source: J Cell Mol Med. 2021 Oct 17;25(21):10279–90. doi: 10.1111/jcmm.16966 (PMC8572768; doi:10.1111/jcmm.16966)
Supplement: Supplementary file 1 — Figure S1 [file JCMM-25-10279-s001.docx]

**Supplementary Figures**

**
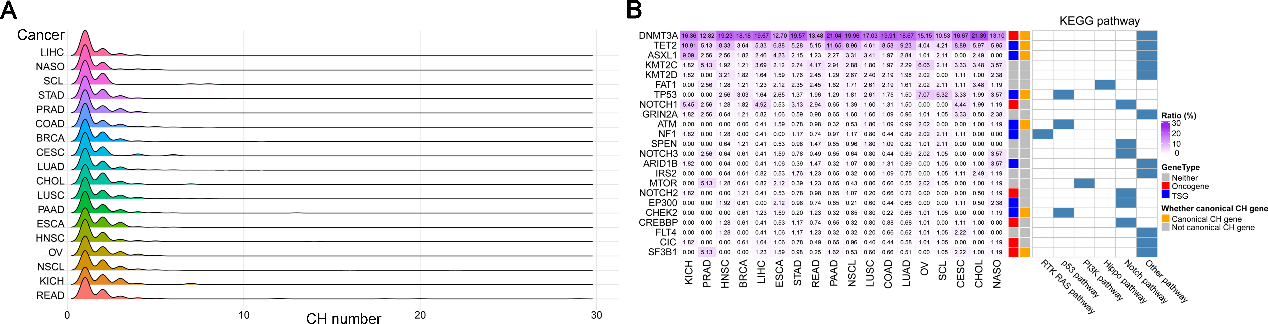
**

**Figure S1** Characterization of CH mutations in the Chinese pan-cancer cohort. **(A)** Distribution of patients with different numbers of CH mutations in each cancer type. **(B)** Heatmap of the top mutated genes carrying CH in different cancer types. We ranked genes by CH mutation number. The number in the cells indicate “ratio” for each gene in the corresponding cancer type. The “Ratio” represents the percentage of CH mutations in each gene carried by patients of each cancer type and total CH mutations in corresponding cancer type.
